# Bone Marrow-Derived Mesenchymal Stem Cells Differentiate into Cancer-Associated Fibroblasts and Promote Tumor Growth in Renal Cell Carcinoma

**DOI:** 10.3390/cancers18111716

**Published:** 2026-05-25

**Authors:** Hiroyuki Kitano, Ryo Yuge, Hiroyuki Shikuma, Kazuma Yukihiro, Tomoya Hatayama, Yoshinori Nakano, Shinsaku Tasaka, Mai Okazaki, Naofumi Nomura, Ryo Tasaka, Kyosuke Iwane, Yuki Kohada, Shunsuke Miyamoto, Miki Naito, Hidehiko Takigawa, Kohei Kobatake, Yohei Sekino, Shiro Oka, Nobuyuki Hinata

**Affiliations:** 1 Department of Urology, Graduate School of Biomedical and Health Sciences, Hiroshima University, Hiroshima 734-8551, Japan; hshikuma@hiroshima-u.ac.jp (H.S.); kytr1989@hiroshima-u.ac.jp (K.Y.); htomoya1211@hiroshima-u.ac.jp (T.H.); nakanoyo@hiroshima-u.ac.jp (Y.N.); tasashin@hiroshima-u.ac.jp (S.T.); maiok34@hiroshima-u.ac.jp (M.O.); nomuran@hiroshima-u.ac.jp (N.N.); tasakary@hiroshima-u.ac.jp (R.T.); kiwane@hiroshima-u.ac.jp (K.I.); ykohada@hiroshima-u.ac.jp (Y.K.); sm0025@hiroshima-u.ac.jp (S.M.); mikinaito@hiroshima-u.ac.jp (M.N.); kkobatake@hiroshima-u.ac.jp (K.K.); swimming@hiroshima-u.ac.jp (Y.S.); hinata@hiroshima-u.ac.jp (N.H.); 2 Department of Gastroenterology, Graduate School of Biomedical and Health Sciences, Hiroshima University, 1-2-3 Kasumi, Minami-ku, Hiroshima 734-8551, Japan; makapoo@hiroshima-u.ac.jp (R.Y.); hidehiko@hiroshima-u.ac.jp (H.T.); oka4683@hiroshima-u.ac.jp (S.O.); 3 Department of Gastroenterology, Hiroshima Red Cross Hospital and Atomic-Bomb Survivors Hospital, Hiroshima 730-8619, Japan

**Keywords:** renal cell carcinoma, mesenchymal stem cells, cancer-associated fibroblasts, tumor microenvironment, orthotopic xenograft model

## Abstract

**Background**: Tumor–stroma interactions play a critical role in renal cell carcinoma (RCC) progression. Cancer-associated fibroblasts (CAFs) are considered key components of the tumor microenvironment; however, their origin remains controversial. This study aimed to determine whether bone marrow-derived mesenchymal stem cells (MSCs) contribute to CAF-like stromal changes and RCC progression. **Methods**: An orthotopic xenograft mouse model was established using luciferase- and GFP-labeled Caki-1 cells. MSCs labeled with PKH26 were administered intravenously. Tumor growth was evaluated using an in vivo imaging system and tumor volume measurements. Immunohistochemical analyses were performed to assess MSC localization and α-smooth muscle actin (α-SMA) expression. In vitro proliferation and migration assays were conducted using direct and indirect co-culture systems. **Results**: The intravenous administration of MSCs significantly increased tumor growth and bioluminescence intensity in an orthotopic model. The tumor volumes were significantly larger in the MSC-treated versus control group. An immunofluorescence analysis demonstrated partial co-localization of PKH26-labeled MSCs with α-SMA-positive fibroblast-like cells, suggesting acquisition of CAF-like features. Direct co-culture with MSCs significantly enhanced RCC cell proliferation and migration in vitro, whereas culturing in conditioned medium alone did not produce similar effects. **Conclusions**: Exogenously administered bone marrow-derived MSCs may be recruited into RCC tissues and acquire CAF-like features through interactions with tumor cells. These findings suggest that stromal–tumor cell interactions within the tumor microenvironment may contribute to RCC progression and represent a potential therapeutic target.

## Simple Summary

Renal cell carcinoma is a common type of kidney cancer that can become aggressive and spread to other parts of the body. Cancer growth is influenced not only by tumor cells themselves but also by surrounding support cells in the tumor environment. This study investigated whether bone marrow-derived mesenchymal stem cells can enter kidney tumors and transform into cancer-supporting fibroblast-like cells. Using a mouse model and laboratory experiments, the researchers found that these cells migrated into tumors, increased tumor growth, and enhanced cancer cell movement when in direct contact with tumor cells. These findings suggest that certain stem cells from the bone marrow may help create a tumor environment that supports cancer progression. Understanding this process may help researchers develop new treatments that target the tumor environment, in addition to targeting cancer cells directly.

## 1. Introduction

Renal cell carcinoma (RCC) is the most common malignancy of the kidney, and accounts for approximately 2–3% of all adult cancers worldwide [1]. According to global cancer statistics, more than 430,000 new cases of kidney cancer are diagnosed annually along with approximately 180,000 deaths [2]. Despite advances in diagnostic imaging and the development of targeted therapies and immune checkpoint inhibitors, the prognosis of advanced RCC remains poor due to local recurrence and distant metastasis [3]. Therefore, a better understanding of the molecular mechanisms underlying RCC development and progression is essential to identifying novel biomarkers and developing more effective therapeutic strategies.

Tumors consist of malignant cells and a complex tumor microenvironment (TME) that includes the tumor stroma. The tumor stroma consists of various cellular components, such as fibroblasts, smooth muscle cells, vascular cells (including endothelial cells and pericytes), inflammatory cells, and the extracellular matrix (ECM) enriched with cytokines and growth factors [4]. Previous tumor biology studies have demonstrated that tumor growth and metastasis are influenced by the malignant cells themselves as well as by the surrounding stromal components and that cancer cell–stromal cell interactions play an important role in tumor progression [5,6]. In particular, carcinoma-associated fibroblasts (CAFs) play a central role in the TME [7], and reciprocal interactions between CAFs and cancer cells are hypothesized to promote tumor growth and development [8].

In RCC, inhibition of platelet-derived growth factor receptor signaling in CAFs by pharmacological agents has been reported to induce tumor regression, highlighting the important role of CAFs in tumor progression [9]. In colorectal cancer, mesenchymal stem cells (MSCs) are known to migrate into the tumor stroma and differentiate into CAFs, which promote the growth and metastasis of colon cancer [10]. However, the origin and specific role of CAFs within the TME in RCC remain unclear. Therefore, the present study aimed to clarify the origin of CAFs and elucidate their role in the TME of RCC.

## 2. Materials and Methods

### 2.1. Human RCC Cell Lines and Culture Conditions

The human RCC cell line luciferase-transfected Caki-1 was a gift from Dr. Peter Black (Vancouver Prostate Center, University of British Columbia, Vancouver, BC, Canada). Luciferase-transfected Caki-1 cells were cultured under previously described conditions [9]. Cell line identity was confirmed based on information provided by the supplier. All cell lines were confirmed to be free of mycoplasma contamination prior to use.

### 2.2. Culture of Human MSCs

Human MSCs isolated from the iliac crest were provided by Dr. Yasuhiko Kitadai and cultured as previously described [11,12]. In the culture medium, MSCs formed a monolayer of adherent cells with a long spindle-shaped fibroblast-like morphology.

### 2.3. Animals and Transplantation of Tumor Cells and In Vivo Assessment of MSC Migration in Kidney

Female athymic nude BALB/c mice were maintained under specific pathogen-free conditions and used at 8 weeks of age. All animal experiments were approved by the Committee on Animal Experimentation of Hiroshima University. Orthotopic renal tumors were established by injecting luciferase-transfected Caki-1 cells (1 × 10^6^ cells) into the left renal capsule, as previously described [9].

Two weeks after tumor implantation, mice were randomly assigned to the MSC or control group. PKH26-labeled MSCs (1 × 10^6^ cells) suspended in HBSS were intravenously injected via the tail vein in the MSC group, whereas control mice received HBSS alone [10]. Two weeks later, tumors were harvested and analyzed by fluorescence confocal microscopy.

### 2.4. Necropsy and Histological Studies

Mice with orthotopic tumors were euthanized under deep anesthesia using a mixture of medetomidine, midazolam, and butorphanol and their body weights measured. After necropsy, the tumors were excised and weighed. One portion of the tumor was then fixed in a formalin-free immunohistochemical zinc fixative provided as a ready-to-use solution (BD Biosciences, San Jose, CA, USA) at room temperature for 24 h and embedded in paraffin for immunohistochemical analysis.

### 2.5. In Vitro Assessment of Cell Proliferation and Motility

Caki-1 cells were cultured under three conditions: (1) Caki-1 monoculture; (2) Caki-1 cultured in medium supplemented with MSC-conditioned medium (MSC-CM) to represent an indirect co-culture condition; and (3) direct co-culture of Caki-1 cells with MSCs (5 × 10^3^ cells/well). Comparisons were performed between conditions 1 and 2 as well as conditions 1 and 3. The CM derived from MSCs was collected to evaluate the potential paracrine effects of MSCs on KM12SM cells. Briefly, MSCs (1.0 × 10^6^ cells) were seeded in a 100-mm culture dish. After 48 h of incubation, the culture medium was replaced with 20 mL of DMEM containing 0.5% FBS, and the cells were further incubated for 24 h. The CM was then collected, passed through a sterile filter, aliquoted, and stored at −20 °C until use. DMEM supplemented with 0.5% FBS was used as the control medium. For the proliferation assay, Caki-1 cells (5 × 10^3^ cells/well) were seeded in 24-well ImageLock plates (Essen Bioscience, Ann Arbor, MI, USA) containing RPMI 1640 medium supplemented with 0.5% FBS and cultured alone or with MSCs (5 × 10^3^ cells/well). Bright-field images were obtained using a label-free, high-content, time-lapse imaging system (IncuCyte Zoom; Essen Bioscience). Cell confluence was automatically quantified as a percentage over 4 days using IncuCyte software (version 2015A Rev1; Essen Bioscience), and growth curves were generated. All experiments were performed in triplicate.

Cell migration was evaluated using a scratch wound assay. Caki-1 cells were seeded at a density of 1 × 10^5^ cells/well on 96-well ImageLock plates (Essen Bioscience) coated with 100 μg/mL Matrigel (cat. no. 354234; BD Biosciences). Matrigel was diluted in RPMI 1640 medium containing 0.5% FBS and coated at 37 °C for 24 h. The cells were cultured in RPMI 1640 supplemented with 0.5% FBS alone or with MSCs (1 × 10^5^ cells/well) until the cell monolayer reached approximately 100% confluence.

Scratch wounds were created using a 96-pin wound marker of the IncuCyte system. After wounding, the detached cells were removed by washing twice with phosphate-buffered saline (PBS). Images were automatically captured every 3 h over 2 days using IncuCyte software (version 2015A Rev1; Essen Bioscience) under an inverted microscope equipped with phase and excitation light-emitting diode illumination.

Relative wound density was automatically calculated using IncuCyte software. The relative wound density represents the percentage density of cells within the wound region relative to the surrounding cell region, with an initial value of approximately 5%. All experiments were performed in triplicate [13].

### 2.6. Immunofluorescence Staining

Frozen specimens cut into 8-mm sections or cells cultured on glass slides were fixed for 15 min in 4% paraformaldehyde and PBS. The slides were blocked briefly in protein blocking solution, incubated overnight at 4 °C with the Fab fragment of anti-mouse immunoglobulin G to block endogenous immunoglobulins, and incubated overnight at 4 °C with anti-α-SMA (1:400). The slides were washed with PBS and incubated for 1 h at room temperature with Alexa Fluor 488-labeled secondary antibody (1:600). Nuclear counterstaining with 4ʹ,6-diamidino-2-phenylindole was performed for 10 min, and mounting medium was placed on each specimen with a glass coverslip. All α-SMA-positive cells were identified by green fluorescence, whereas PKH26 in MSCs was detected by red fluorescence. The co-localization of PKH26 and α-SMA was detected by double immunofluorescence staining [10].

### 2.7. Immunohistochemical Staining

Immunohistochemical staining was performed as previously described [9] with the dilution ratios of the specified primary antibodies.

### 2.8. Confocal Microscopy

Confocal fluorescence images were collected using 10×, 20×, and 40× objective lenses on a Zeiss LSM laser scanning microscopy system (Carl Zeiss, Jena, Germany) equipped with a motorized axioplan microscope, argon laser (458/477/488/514 nm, 30 mW), HeNe laser (543 nm, 1 mW), HeNe laser (633 nm, 5 mW), LSM 510 control and image acquisition software, and appropriate filters (Chroma Technology Corp., Brattleboro, VT, USA). Confocal images were exported to Adobe Photoshop (version 21.0; Adobe Inc., San Jose, CA, USA), and montages were prepared for publication.

### 2.9. Statistical Analysis

The statistical analyses were performed using GraphPad Prism (v.11.0; GraphPad Software, San Diego, CA, USA). Data are presented as the mean ± standard deviation (SD). Normality of data distribution was assessed prior to parametric analyses. Intergroup comparisons were performed using Student’s *t*-test or one-way analysis of variance, as appropriate. Values of *p* < 0.05 were considered statistically significant.

## 3. Results

### 3.1. Caki-1 Orthotopic Primary Tumor Growth

**Figure 1:**
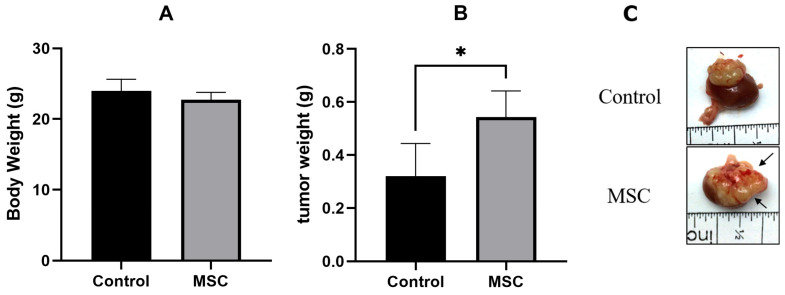
Effects of mesenchymal stem cell (MSC) administration on body weight and tumor growth in an orthotopic renal cell carcinoma model. (**A**) Body weights of mice in the control and MSC-treated groups at the time of necropsy. No significant intergroup differences were noted. (**B**) Tumor weights of orthotopically implanted renal tumors. Tumors were significantly heavier in the MSC-treated versus control group. (**C**) Representative images of excised orthotopic tumors from the control and MSC-treated groups. Data are presented as the mean ± standard deviation. * *p* < 0.05.

On day 14 after tumor cell transplantation, five mice each remained in the control and MSC-treated groups. After the injection of PKH26-labeled MSCs into the tail veins of the tumor-bearing mice, one mouse in the control group died before day 28. Consequently, four and five mice remained in the control and MSC-treated groups, respectively, on day 28. To evaluate the effects of intravenously administered MSCs, we measured the body weights of the host mice and the weights of the orthotopically implanted tumors. No significant intergroup differences in body weight were noted (*p* = 0.257; Figure 1A). However, the excised tumors were significantly larger in the MSC than control group (*p* = 0.027; Figure 1B, C).

**Figure 2:**
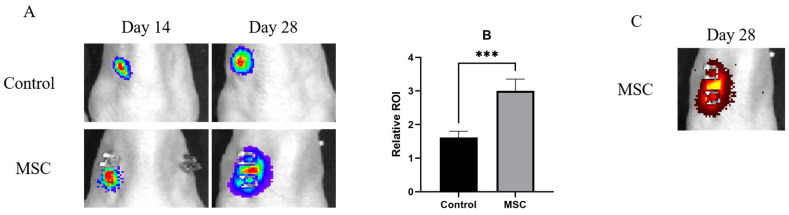
In vivo imaging analysis of tumor growth and mesenchymal stem cell (MSC) localization. (**A**) Representative bioluminescence images of orthotopic tumors derived from luciferase-expressing Caki-1 cells. (**B**) Quantitative analysis of relative luciferase signal intensity demonstrating significantly higher tumor activity in the MSC-treated versus control group. (**C**) Detection of PKH26 fluorescence signal derived from intravenously injected MSCs. Fluorescence was observed only in the MSC-treated group. Data are presented as the mean ± standard deviation. *** *p* < 0.001.

We also monitored the luciferase activity of the Caki-1 cells using an IVIS imaging system (PerkinElmer, Waltham, MA, USA) (Figure 2A). Luciferase activity was significantly higher in mice in the MSC versus control group (Figure 2B; *p* = 0.0002). We also measured PKH26 fluorescence, which was used to label the MSCs, using an IVIS imaging system. No detectable fluorescence was observed in the control group, whereas PKH26 fluorescence was detected in the MSC-treated group (Figure 2C). The mean relative fluorescence intensity in the MSC-treated group was 184.25 (SD, 32.9).

### 3.2. MSC-Derived CAF-like Cells in Orthotopic Tumors

**Figure 3:**
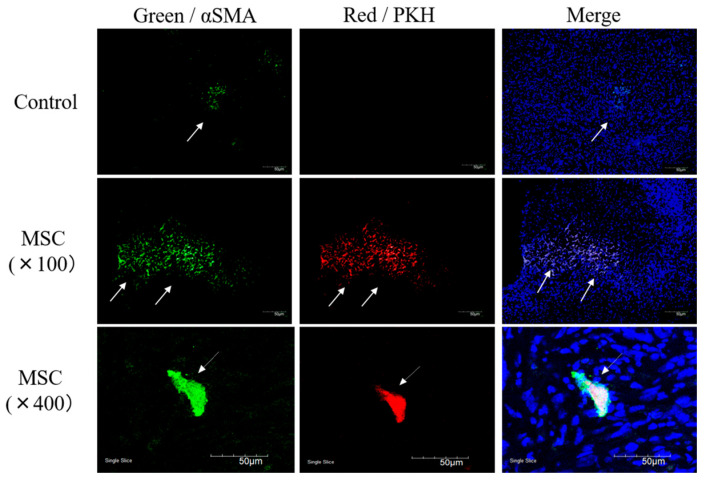
Localization of PKH26-labeled mesenchymal stem cells (MSCs) and expression of α-smooth muscle actin (α-SMA) in orthotopic tumors. Immunofluorescence staining of tumor sections. Green: α-SMA; red: PKH26-labeled MSCs; blue: 4′,6-diamidino-2-phenylindole nuclear staining. In the MSC-treated group, PKH26-positive cells were detected within the tumor stroma and partially co-localized with α-SMA-positive fibroblast-like cells (arrows). The merged images indicate the differentiation of MSCs into cancer-associated fibroblast (CAF)-like cells within the tumor microenvironment. Images were obtained at ×100 and ×400 magnification. Scale bar = 50 μm.

Two weeks after the orthotopic transplantation of Caki-1 cells, PKH26-labeled MSCs were injected into the tail veins of tumor-bearing mice. Immunofluorescence double staining demonstrated that PKH26-labeled MSCs (red) were localized within the tumor stroma and co-expressed α-SMA (green) (Figure 3). Merged images confirmed the co-localization of PKH26 and α-SMA, indicating that the injected MSCs differentiated into CAF-like cells within the TME.

### 3.3. Effects of MSC Co-Culturing on In Vitro Caki-1 Cell Proliferation and Migration

**Figure 4:**
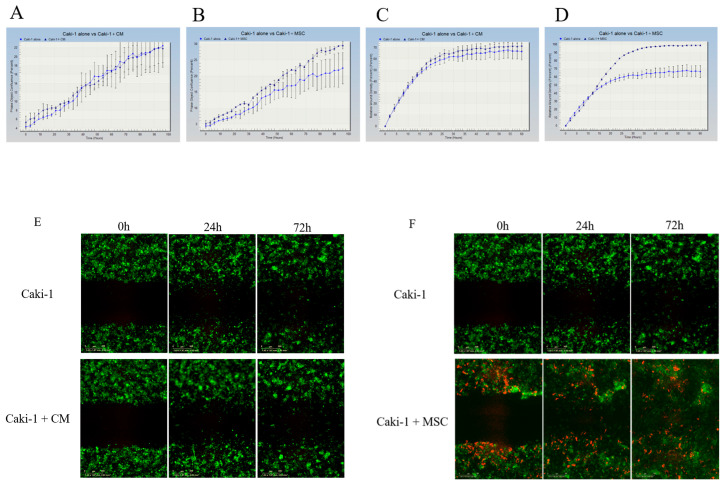
Effects of mesenchymal stem cell (MSC) co-culture on in vitro Caki-1 cell proliferation and migration. Red fluorescence represents PKH26-labeled MSCs. (**A**) Proliferation curves of Caki-1 cells cultured alone or with MSC-conditioned medium (MSC-CM). No significant difference in proliferation was observed. (**B**) Proliferation curves of Caki-1 cells cultured alone or directly with MSCs, showing significantly increased proliferation under direct co-culture conditions. (**C**) Migration analysis using a scratch wound assay comparing Caki-1 cells cultured alone and with MSC-CM. No significant differences were observed. (**D**) Migration analysis showing significantly enhanced migration of Caki-1 cells under direct co-culturing with MSCs. (**E**) Representative time-lapse images of scratch wound closure in Caki-1 cells cultured alone or with MSC-CM (0 h, 24 h, and 72 h). (**F**) Representative time-lapse images of scratch wound closure during direct co-culturing with MSCs demonstrating MSC migration into the wound area followed by Caki-1 cell migration.

To evaluate the effects of MSCs on the biological behavior of renal cancer cells, Caki-1 cells were cultured alone or co-cultured with MSCs using indirect (CM) or direct co-culture systems. Cell proliferation was monitored by real-time imaging. No significant difference in proliferation was observed between Caki-1 cells cultured alone versus in MSC-CM (Figure 4A; *p* = 0.7657). However, Caki-1 cell proliferation significantly increased upon direct co-culturing with MSCs (Figure 4B; *p* = 0.0484).

Cell migration was evaluated using the scratch wound assay. No significant difference in migration was observed between Caki-1 cells cultured alone versus those co-cultured with MSC-CM (Figure 4C; *p* = 0.587). In contrast, Caki-1 cell migration was significantly enhanced under direct co-culture with MSCs versus culturing alone (Figure 4D; *p* = 0.0169). Time-lapse imaging up to 72 h demonstrated that Caki-1 cell migration was limited when cultured in MSC-CM (Figure 4E). In contrast, under direct co-culture conditions, MSCs first migrated to the scratch area, followed by Caki-1 cells (Figure 4F). These findings suggest that MSCs facilitate Caki-1 cell migration toward the wound area. Collectively, these results indicate that the direct interaction between MSCs and cancer cells promotes Caki-1 cell migratory capacity.

### 3.4. Accumulation of CAF-like Cells at Tumor Margin Following MSC Injection

**Figure 5:**
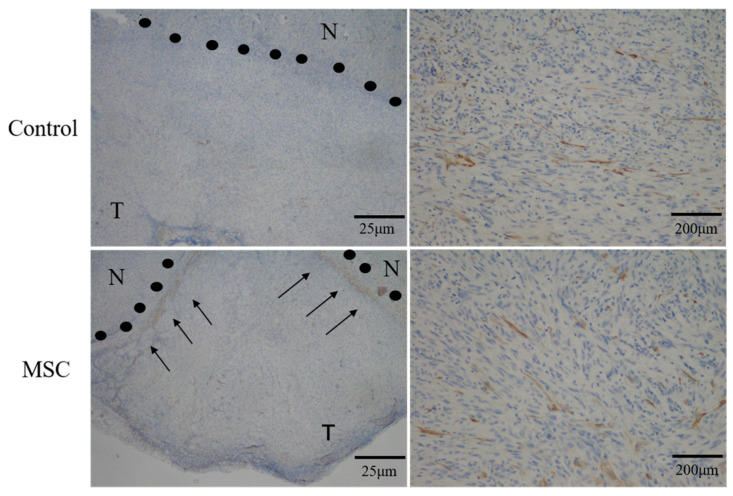
Cancer-associated fibroblast (CAF) accumulation at the tumor margin in mesenchymal stem cell (MSC)-treated group. Immunohistochemical staining demonstrated clustering of α-smooth muscle actin (α-SMA)-positive CAF-like cells at the tumor margin and invasive front in tumors from MSC-treated mice, whereas control tumors showed only sparse α-SMA-positive cells.

Immunohistochemical staining of the resected orthotopic tumors was performed. In tumors from mice that received tail vein injection of MSCs, clusters of α-SMA-positive cells, indicative of CAF-like cells, were observed at the tumor–adjacent normal kidney tissue interface. In contrast, in the control tumors, α-SMA-positive cells were not accumulated at the tumor margin but rather sparsely distributed throughout the tumor (Figure 5).

## 4. Discussion

The co-culturing of breast cancer cell lines with human MSCs reportedly induces the differentiation of MSCs into CAFs [14]. Similarly, the prolonged exposure of human MSCs to breast cancer cell lines promotes the acquisition of CAF-like phenotypes that contribute to angiogenesis and tumor growth within the TME [15]. Furthermore, MSCs reportedly migrate to various tumor types, including melanoma [16], glioma [17,18], and colorectal [10,19], pancreatic [20,21], and breast [22,23] cancers. However, the origin of CAFs in RCC has not yet been clearly elucidated [24,25]; moreover, their role in the TME remains poorly characterized compared with that in other malignancies. In the present study, we established an orthotopic xenograft mouse model using a human RCC cell line and intravenously injected human MSCs into the tail veins. Injected MSCs were detected within orthotopically implanted tumors, and tumor growth was enhanced in the MSC-treated group. These findings suggest that exogenously administered bone marrow-derived MSCs may be recruited into RCC tissues and acquire CAF-like features within the TME. Furthermore, in vitro co-culturing experiments demonstrated that RCC cells migrated after MSC movement, suggesting that MSCs may guide cancer cell migration. These observations indicate that interactions between MSCs and RCC cells may contribute to tumor progression.

RCC presents significant clinical challenges because many patients exhibit tumor heterogeneity and metastasis at the time of diagnosis [26]. The TME is increasingly recognized as a critical factor in RCC progression, therapeutic resistance, and metastasis [27]. Among the stromal components of the TME, CAFs support tumor progression by remodeling the ECM, promoting angiogenesis, and exerting immunosuppressive effects [28].

CAFs originate from diverse cellular sources in RCC. Fibroblasts present in the tumor stroma, which are mesenchymal cells derived from organ development, represent a potential precursor population of CAFs. Several other cell types are believed to differentiate into CAFs, including endothelial cells, epithelial cells, vascular smooth muscle cells, pericytes, adipocytes, and tumor cells undergoing epithelial–mesenchymal transition (EMT) [29,30]. Although the differentiation of MSCs into CAFs has been reported for several malignancies, whether MSCs contribute to CAF generation in RCC remains unclear [24,25]. Our results suggest that MSCs migrate to RCC tumors and may differentiate into CAFs.

CAFs are key regulators of EMT, a process that plays a crucial role in tumor invasion, metastasis, and therapeutic resistance [31]. CAFs secrete various cytokines and chemokines, including interleukin-6, osteopontin, hepatocyte growth factor, CXCL12, and insulin-like growth factor-1. These secreted factors influence RCC cell behavior and initiate and sustain the EMT, a central process in tumor metastasis. They activate several oncogenic signaling pathways, including JAK/STAT3, PI3K/AKT/mTOR, and MAPK signaling, and the downstream activation of pathways, such as PI3K/AKT and RAS/MAPK, promotes cancer cell proliferation, survival, and migration [32,33].

As RCC progresses, the number of CAFs increases, and the TME becomes enriched in stromal components [34]. Although our study did not specifically investigate the soluble factors secreted by tumor cells or CAFs, real-time observations using the IncuCyte system revealed that CAFs promote the migration of RCC cells during co-culturing. These findings suggest that dynamic interactions between CAF-like stromal cells and tumor cells may contribute to tumor growth and progression. Consistent with these in vitro findings, our in vivo orthotopic xenograft model demonstrated that intravenously injected MSCs within the tumor tissue were associated with enhanced tumor growth.

This study had several limitations, including a relatively small sample size and lack of direct evidence demonstrating MSC differentiation into CAFs. Because CAF identification in this study was primarily based on α-SMA expression, additional CAF markers and lineage-tracing approaches will be necessary to definitively confirm MSC differentiation into CAFs. Further studies are required to elucidate the mechanisms underlying MSC–tumor interactions in RCC.

Taken together, our findings suggest that exogenously administered MSCs may be recruited into RCC tissues and dynamically interact with tumor cells within the TME. Real-time imaging analysis demonstrated that CAF-like stromal cells may promote RCC cell migration, suggesting that such cellular interactions may contribute to tumor progression. These results highlight the potential importance of stromal–tumor cell interactions in shaping the TME and promoting RCC growth.

In the present co-culture experiments, individual cell populations were not separately tracked. Therefore, the contribution of MSC proliferation or migration to wound closure cannot be completely excluded. In addition, differences in proliferation kinetics between MSCs and RCC cells may influence the interpretation of the co-culture assays. Future studies using lineage-tracing or cell-specific labeling approaches will be necessary to more precisely define the contribution of each cell type.

## 5. Conclusions

In conclusion, our study findings suggest that MSCs migrate to RCC tumors and potentially contribute to the formation of CAF-like stromal cells within the TME. In addition, real-time imaging revealed that stromal cells may guide RCC cell migration, indicating that dynamic interactions between tumor cells and stromal components may play an important role in tumor progression. These findings provide new insights into the role of MSC-derived stromal cells in RCC and highlight the TME as a potential target for future therapeutic strategies.

## Data Availability

The datasets generated and/or analyzed in the current study are available from the corresponding author upon reasonable request.

## Abbreviations

The following abbreviations are used in this manuscript:

RCC	Renal cell carcinoma
CAFs	Cancer-associated fibroblasts
MSCs	Mesenchymal stem cells
alpha-SMA	alpha-smooth muscle actin
TME	Tumor microenvironment
ECM	Extracellular matrix
FBS	Fetal bovine serum
DMEM	Dulbecco’s Modified Eagle’s medium
HBSS	Hanks’ balanced salt solution
MSC-CM	MSC-conditioned medium
PBS	Phosphate-buffered saline
SD	Standard deviation
EMT	Epithelial–mesenchymal transition
